# 2-(4-Ferrocenylphen­yl)-4,4,5,5-tetra­methyl-1,3,2-dioxaborolane

**DOI:** 10.1107/S1600536808035228

**Published:** 2008-11-08

**Authors:** Peter D. W. Boyd, J. D. Paauwe

**Affiliations:** a Department of Chemistry, The University of Auckland, Private Bag 92019, Auckland, New Zealand

## Abstract

In the title compound,, [Fe(C_5_H_5_)(C_17_H_20_BO_2_)], the two near parallel cyclo­penta­dienyl rings of the ferrocene group are eclipsed. The benzene ring is tilted with respect to the attached cyclo­penta­diene ring by 17.0 (1)° and by 24.2 (1)° with respect to the dioxaborolane ring. The mol­ecules assemble in the crystal *via* C—H⋯π inter­actions between the cyclo­penta­dienyl H atoms and the benzene and cyclo­penta­dienyl rings of neighbouring mol­ecules.

## Related literature

For the related tris­(4-ferrocenylphen­yl)boroxine benzene solvate, see: Makarov *et al.* (2004 ▶). For other related structures, see: Anderson *et al.* (2003 ▶); Nyamori & Bala (2008 ▶). For related literature, see: Leclerc *et al.* (2003 ▶).
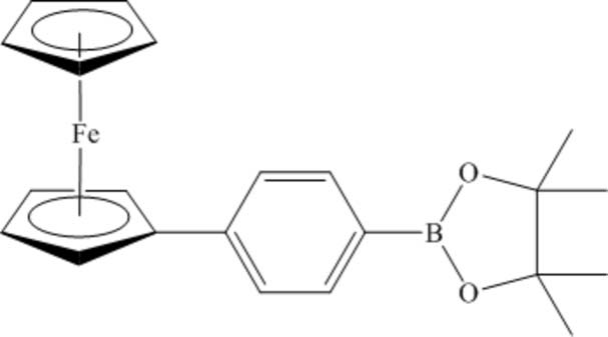


## Experimental

### 

#### Crystal data

[Fe(C_5_H_5_)(C_17_H_20_BO_2_)]
*M* *_r_* = 388.08
Monoclinic, 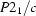

*a* = 12.4439 (3) Å
*b* = 12.9832 (3) Å
*c* = 13.0728 (3) Å
β = 117.126 (1)°
*V* = 1879.75 (8) Å^3^
*Z* = 4
Mo *K*α radiation
μ = 0.82 mm^−1^
*T* = 89 (2) K
0.37 × 0.37 × 0.20 mm

#### Data collection

Bruker SMART APEXII CCD diffractometer
Absorption correction: multi-scan (*SADABS*; Sheldrick, 1996 ▶) *T*_min_ = 0.717, *T*_max_ = 0.849
23134 measured reflections
4442 independent reflections
3984 reflections with *I* > 2σ(*I*)
*R*_int_ = 0.027

#### Refinement

*R*[*F* ^2^ > 2σ(*F* ^2^)] = 0.031
*wR*(*F* ^2^) = 0.085
*S* = 1.03
4442 reflections
239 parameters
H-atom parameters constrained
Δρ_max_ = 0.86 e Å^−3^
Δρ_min_ = −0.31 e Å^−3^

### 

Data collection: *APEX2* (Bruker, 2006 ▶); cell refinement: *SAINT* (Bruker, 2006 ▶); data reduction: *SAINT*; program(s) used to solve structure: *SHELXS97* (Sheldrick, 2008 ▶); program(s) used to refine structure: *SHELXL97* (Sheldrick, 2008 ▶); molecular graphics: *ORTEPIII* (Burnett & Johnson, 1996 ▶) and *Mercury* (Macrae *et al.*, 2006 ▶); software used to prepare material for publication: *WinGX* (Farrugia, 1999 ▶) and *publCIF* (Westrip, 2008 ▶).

## Supplementary Material

Crystal structure: contains datablocks global, I. DOI: 10.1107/S1600536808035228/hg2435sup1.cif

Structure factors: contains datablocks I. DOI: 10.1107/S1600536808035228/hg2435Isup2.hkl

Additional supplementary materials:  crystallographic information; 3D view; checkCIF report

## Figures and Tables

**Table 1:** Hydrogen-bond geometry (Å, °)

*D*—H⋯*A*	*D*—H	H⋯*A*	*D*⋯*A*	*D*—H⋯*A*
C6—H6⋯C12^i^	0.93	2.86	3.6302 (19)	141
C5—H5⋯C6^ii^	0.93	2.62	3.538 (2)	168

Symmetry codes: (i) 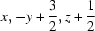; (ii) 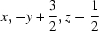.

**Fig. 1.**
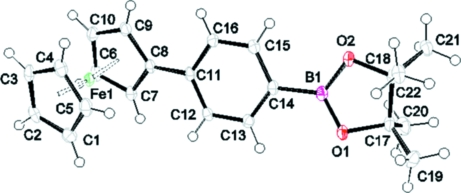
Structure of (I) showing 50% probability displacement ellipsoids for non-H atoms.

**Fig. 2.**
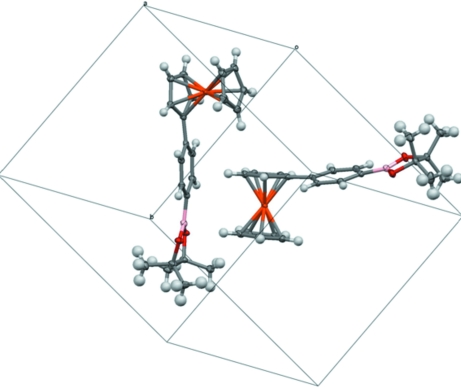
The crystal packing of (I) showing C—H..π interactions between adjacent molecules. [Symmetry codes: x,y,z and 1 - x,1 - y,1 - z]

## supplementary crystallographic information

### Comment

The title compound, (I), was prepared from the reaction of lithiated 4-bromophenylferrocene with 2-isopropoxy-4,4,5,5-tetramethyl-1,3,2-dioxoborolane in tetrahydrofuran. Unlike the related tris(4-ferrocenylphenyl)boroxine benzene solvate (Makarov *et al.* (2004), the 2-(4-ferrocenyl-phenyl)-4,4,5,5-tetramethyl-1,3,2-dioxaborolane is monomeric (Fig. 1). The two cyclopentadienyl rings are nearly eclipsed, average torsion angle 2.1 (1)°, with a small tilt of the two planes (C1—C5 and C6—C10) of 3.5 (1)°. The distances of the iron atom to the ring centroids were 1.6514 (2)Å and 1.6475 (2) Å respectively. The phenyl ring is tilted by 17.0 (1)° with respect to the (C6—C10) plane. This value is slightly higher than that observed in similar structures (Anderson *et al.* (2003), Nyamori and Bala (2008)). The dioxaborolane ring is in a half-chair conformation, with an O1—C17—C18—O2 torsion angle of 24.2 (1)°. The BO_2_ group is rotated away from the plane of the phenyl ring system by 11.1 (2)°, and the angle between the dioxaborolane ring and the phenylplane is 9.9 (1)°. The molecules pack in the crystal, (Fig. 2), with C—H···π interactions between cyclopentadienyl hydrogen atoms and the phenyl and cyclopentadienyl rings of neighbouring molecules, Table 1.

### Experimental

To a solution of 4-bromophenyl ferrocene (0.2 g, 0.59 mmol) in dry THF (10 mL) stirred at -78°C under nitrogen was added dropwise a solution of n-BuLi 2.5*M* in hexane (0.51 ml, 0.88 mmol).The mixture was then stirred at -78 °C for 20 minutes. Then 2-isopropoxy-4,4,5,5-tetramethyl-1,3,2-dioxoborolane (0.18 ml, 0.88 mmol) was added, the stirring was kept at -78°C for 2 h and the mixture allowed to warm to -35 C and stir for 1 h and then warmed to room temperature. The reaction mixture was then poured into water and extracted with diethyl ether (2 *x* 25 ml). The combined organic layers were washed with brine and dried with Na_2_SO_4_. The solvent removed under reduced pressure and purified by column chromatography (SiO_2_, Hexane/DCM =2/1) to give the pure compound 2-(4-ferrocenyl-phenyl)-4,4,5,5-tetramethyl-1,3,2-dioxaborolane (0.052 g, 0.129 mmol, 23%) as an orange solid. FAB-MS (C_22_H_25_^10^BFeO_2_) 387.13293 (C_22_H_25_^11^BFeO_2_) 388.12934. ^1^H NMR (CDCl_3_, 300 MHz) δ 1.36 (CH_3_, s 12H), 4.02 (CpH, s, 5H), 4.33 (CpH, t, J=1.84, 2H), 4.68 (CpH, t, J =1.85 2H), 7.46 (ArH, d, J=8.3 2H), 7.72 (ArH, d, J=8.3 2H) p.p.m..

### Refinement

Hydrogen atoms were placed in calculated positions and refined using the riding model [C—H 0.93–0.97 Å), with *U*_iso_(H) = 1.2 and 1.5 times *U*_eq_(C) for aromatic and alkyl groups respectively. In the case of the methyl groups protons were rotated to fit the H-atom positions to the observed electron density.

### Crystal data

**Table:**

[Fe(C_5_H_5_)(C_17_H_20_BO_2_)]	*F*_000_ = 816
*M**_r_* = 388.08	*D*_x_ = 1.371 Mg m^−^^3^
Monoclinic, *P*2_1_/*c*	Mo *K*α radiation λ = 0.71073 Å
Hall symbol: -P 2ybc	Cell parameters from 9781 reflections
*a* = 12.4439 (3) Å	θ = 1.8–27.8º
*b* = 12.9832 (3) Å	µ = 0.82 mm^−^^1^
*c* = 13.0728 (3) Å	*T* = 89 (2) K
β = 117.126 (1)º	Block, orange
*V* = 1879.75 (8) Å^3^	0.37 × 0.37 × 0.2 mm
*Z* = 4	

### Data collection

**Table:**

Bruker SMART APEXII CCD diffractometer	4442 independent reflections
Radiation source: fine-focus sealed tube	3984 reflections with *I* > 2σ(*I*)
Monochromator: graphite	*R*_int_ = 0.027
*T* = 89(2) K	θ_max_ = 27.8º
ω scans	θ_min_ = 1.8º
Absorption correction: Multi-scan(SADABS; Sheldrick, 1996)	*h* = −16→14
*T*_min_ = 0.717, *T*_max_ = 0.849	*k* = 0→17
23134 measured reflections	*l* = 0→17

### Refinement

**Table:**

Refinement on *F*^2^	Secondary atom site location: difference Fourier map
Least-squares matrix: full	Hydrogen site location: inferred from neighbouring sites
*R*[*F*^2^ > 2σ(*F*^2^)] = 0.031	H-atom parameters constrained
*wR*(*F*^2^) = 0.085	*w* = 1/[σ^2^(*F*_o_^2^) + (0.0491*P*)^2^ + 1.0943*P*] where *P* = (*F*_o_^2^ + 2*F*_c_^2^)/3
*S* = 1.04	(Δ/σ)_max_ = 0.001
4442 reflections	Δρ_max_ = 0.86 e Å^−^^3^
239 parameters	Δρ_min_ = −0.31 e Å^−^^3^
Primary atom site location: structure-invariant direct methods	Extinction correction: none

### Special details

**Table:**

Geometry. All e.s.d.'s (except the e.s.d. in the dihedral angle between two l.s. planes) are estimated using the full covariance matrix. The cell e.s.d.'s are taken into account individually in the estimation of e.s.d.'s in distances, angles and torsion angles; correlations between e.s.d.'s in cell parameters are only used when they are defined by crystal symmetry. An approximate (isotropic) treatment of cell e.s.d.'s is used for estimating e.s.d.'s involving l.s. planes.
Refinement. Refinement of *F*^2^ against ALL reflections. The weighted *R*-factor *wR* and goodness of fit *S* are based on *F*^2^, conventional *R*-factors *R* are based on *F*, with *F* set to zero for negative *F*^2^. The threshold expression of *F*^2^ > σ(*F*^2^) is used only for calculating *R*-factors(gt) *etc*. and is not relevant to the choice of reflections for refinement. *R*-factors based on *F*^2^ are statistically about twice as large as those based on *F*, and *R*- factors based on ALL data will be even larger.

### Fractional atomic coordinates and isotropic or equivalent isotropic displacement parameters (Å^2^)

**Table:**

	*x*	*y*	*z*	*U*_iso_*/*U*_eq_	
Fe1	0.244744 (18)	0.661122 (14)	0.209954 (16)	0.01545 (8)	
O2	0.36539 (9)	1.21833 (8)	−0.03562 (9)	0.0207 (2)	
O1	0.16554 (9)	1.19391 (8)	−0.16076 (9)	0.0195 (2)	
C11	0.26427 (12)	0.89643 (10)	0.14427 (11)	0.0157 (3)	
C14	0.26540 (13)	1.05937 (10)	0.00034 (12)	0.0167 (3)	
C18	0.33635 (13)	1.29767 (11)	−0.12386 (12)	0.0187 (3)	
C2	0.16209 (14)	0.52057 (11)	0.16048 (14)	0.0231 (3)	
H2	0.1105	0.4901	0.1854	0.028*	
C16	0.37235 (12)	0.94523 (10)	0.16343 (12)	0.0178 (3)	
H16	0.4447	0.9239	0.2241	0.021*	
C7	0.15815 (13)	0.78484 (11)	0.23606 (12)	0.0186 (3)	
H7	0.0789	0.8050	0.1895	0.022*	
C13	0.15859 (13)	1.00753 (10)	−0.02096 (12)	0.0172 (3)	
H13	0.0868	1.0269	−0.0836	0.021*	
C8	0.26304 (13)	0.81901 (11)	0.22597 (12)	0.0167 (3)	
C9	0.36571 (13)	0.76831 (11)	0.31467 (12)	0.0187 (3)	
H9	0.4454	0.7758	0.3281	0.022*	
C6	0.19744 (14)	0.71467 (11)	0.33018 (12)	0.0209 (3)	
H6	0.1480	0.6811	0.3556	0.025*	
B1	0.26500 (15)	1.15783 (11)	−0.06779 (14)	0.0174 (3)	
C15	0.37260 (13)	1.02504 (11)	0.09282 (12)	0.0184 (3)	
H15	0.4453	1.0564	0.1071	0.022*	
C5	0.23381 (15)	0.61510 (11)	0.05503 (13)	0.0243 (3)	
H5	0.2370	0.6572	−0.0011	0.029*	
C21	0.39377 (14)	1.39832 (11)	−0.06503 (13)	0.0238 (3)	
H21A	0.3680	1.4143	−0.0079	0.036*	
H21B	0.3695	1.4526	−0.1210	0.036*	
H21C	0.4801	1.3916	−0.0290	0.036*	
C4	0.33403 (14)	0.56821 (11)	0.14749 (13)	0.0226 (3)	
H4	0.4144	0.5745	0.1628	0.027*	
C10	0.32509 (14)	0.70475 (11)	0.37867 (12)	0.0208 (3)	
H10	0.3735	0.6639	0.4413	0.025*	
C12	0.15769 (12)	0.92783 (10)	0.04947 (12)	0.0169 (3)	
H12	0.0854	0.8949	0.0335	0.020*	
C17	0.19486 (13)	1.29760 (11)	−0.18488 (13)	0.0201 (3)	
C22	0.39120 (14)	1.26270 (11)	−0.20117 (14)	0.0235 (3)	
H22A	0.4761	1.2510	−0.1555	0.035*	
H22B	0.3790	1.3150	−0.2573	0.035*	
H22C	0.3530	1.2000	−0.2395	0.035*	
C3	0.29014 (14)	0.50988 (11)	0.21282 (14)	0.0228 (3)	
H3	0.3369	0.4715	0.2783	0.027*	
C20	0.14060 (15)	1.37292 (13)	−0.13159 (16)	0.0294 (3)	
H20A	0.0554	1.3605	−0.1625	0.044*	
H20B	0.1539	1.4422	−0.1489	0.044*	
H20C	0.1783	1.3635	−0.0498	0.044*	
C19	0.13736 (16)	1.31085 (15)	−0.31429 (14)	0.0312 (4)	
H19A	0.1653	1.2574	−0.3471	0.047*	
H19B	0.1593	1.3768	−0.3324	0.047*	
H19C	0.0511	1.3068	−0.3453	0.047*	
C1	0.12727 (14)	0.58627 (12)	0.06311 (13)	0.0249 (3)	
H1	0.0489	0.6067	0.0136	0.030*	

### Atomic displacement parameters (Å^2^)

**Table:**

	*U*^11^	*U*^22^	*U*^33^	*U*^12^	*U*^13^	*U*^23^
Fe1	0.02072 (12)	0.00975 (11)	0.01600 (12)	−0.00098 (7)	0.00849 (9)	0.00033 (7)
O2	0.0219 (5)	0.0155 (5)	0.0231 (5)	−0.0012 (4)	0.0087 (4)	0.0059 (4)
O1	0.0234 (5)	0.0134 (5)	0.0205 (5)	−0.0019 (4)	0.0090 (4)	0.0031 (4)
C11	0.0225 (6)	0.0095 (5)	0.0171 (6)	−0.0009 (5)	0.0106 (5)	−0.0026 (5)
C14	0.0228 (7)	0.0119 (6)	0.0174 (6)	0.0003 (5)	0.0110 (5)	−0.0002 (5)
C18	0.0234 (7)	0.0130 (6)	0.0206 (7)	0.0000 (5)	0.0107 (6)	0.0037 (5)
C2	0.0296 (8)	0.0134 (6)	0.0292 (8)	−0.0063 (6)	0.0159 (6)	−0.0055 (6)
C16	0.0196 (6)	0.0135 (6)	0.0192 (6)	0.0003 (5)	0.0078 (5)	−0.0001 (5)
C7	0.0243 (7)	0.0137 (6)	0.0202 (7)	−0.0002 (5)	0.0122 (6)	−0.0005 (5)
C13	0.0214 (7)	0.0144 (6)	0.0155 (6)	0.0007 (5)	0.0080 (5)	−0.0011 (5)
C8	0.0235 (7)	0.0105 (6)	0.0170 (6)	−0.0017 (5)	0.0099 (5)	−0.0018 (5)
C9	0.0232 (7)	0.0140 (6)	0.0176 (6)	−0.0024 (5)	0.0081 (6)	−0.0015 (5)
C6	0.0310 (8)	0.0147 (6)	0.0214 (7)	−0.0024 (5)	0.0158 (6)	0.0000 (5)
B1	0.0228 (8)	0.0131 (7)	0.0182 (7)	−0.0004 (5)	0.0111 (6)	−0.0004 (5)
C15	0.0207 (6)	0.0142 (6)	0.0215 (7)	−0.0014 (5)	0.0106 (6)	−0.0008 (5)
C5	0.0386 (9)	0.0156 (7)	0.0207 (7)	−0.0018 (6)	0.0153 (6)	−0.0020 (5)
C21	0.0284 (7)	0.0160 (7)	0.0263 (7)	−0.0033 (6)	0.0118 (6)	0.0005 (6)
C4	0.0291 (7)	0.0154 (6)	0.0273 (7)	−0.0018 (6)	0.0165 (6)	−0.0046 (6)
C10	0.0305 (8)	0.0149 (6)	0.0153 (6)	−0.0012 (5)	0.0090 (6)	0.0011 (5)
C12	0.0210 (6)	0.0127 (6)	0.0185 (6)	−0.0031 (5)	0.0102 (5)	−0.0032 (5)
C17	0.0246 (7)	0.0128 (6)	0.0241 (7)	0.0002 (5)	0.0122 (6)	0.0038 (5)
C22	0.0303 (8)	0.0157 (6)	0.0308 (8)	−0.0003 (6)	0.0193 (7)	0.0017 (6)
C3	0.0316 (8)	0.0103 (6)	0.0272 (7)	0.0015 (5)	0.0140 (6)	0.0007 (5)
C20	0.0302 (8)	0.0184 (7)	0.0438 (10)	0.0026 (6)	0.0205 (8)	−0.0005 (7)
C19	0.0293 (8)	0.0348 (9)	0.0256 (8)	0.0004 (7)	0.0090 (7)	0.0131 (7)
C1	0.0274 (8)	0.0194 (7)	0.0216 (7)	−0.0019 (6)	0.0057 (6)	−0.0056 (6)

### Geometric parameters (Å, °)

**Table:**

Fe1—C6	2.0357 (14)	C13—C12	1.3885 (19)
Fe1—C3	2.0388 (14)	C13—H13	0.9300
Fe1—C10	2.0431 (14)	C8—C9	1.435 (2)
Fe1—C4	2.0454 (15)	C9—C10	1.4220 (19)
Fe1—C7	2.0477 (14)	C9—H9	0.9300
Fe1—C2	2.0492 (14)	C6—C10	1.422 (2)
Fe1—C9	2.0488 (14)	C6—H6	0.9300
Fe1—C1	2.0512 (15)	C15—H15	0.9300
Fe1—C5	2.0558 (15)	C5—C4	1.419 (2)
Fe1—C8	2.0626 (14)	C5—C1	1.427 (2)
O2—B1	1.3695 (19)	C5—H5	0.9300
O2—C18	1.4636 (16)	C21—H21A	0.9600
O1—B1	1.3626 (19)	C21—H21B	0.9600
O1—C17	1.4663 (16)	C21—H21C	0.9600
C11—C12	1.4007 (19)	C4—C3	1.424 (2)
C11—C16	1.4020 (19)	C4—H4	0.9300
C11—C8	1.4718 (18)	C10—H10	0.9300
C14—C13	1.3998 (19)	C12—H12	0.9300
C14—C15	1.403 (2)	C17—C19	1.516 (2)
C14—B1	1.557 (2)	C17—C20	1.525 (2)
C18—C21	1.519 (2)	C22—H22A	0.9600
C18—C22	1.524 (2)	C22—H22B	0.9600
C18—C17	1.567 (2)	C22—H22C	0.9600
C2—C3	1.426 (2)	C3—H3	0.9300
C2—C1	1.426 (2)	C20—H20A	0.9600
C2—H2	0.9300	C20—H20B	0.9600
C16—C15	1.3887 (19)	C20—H20C	0.9600
C16—H16	0.9300	C19—H19A	0.9600
C7—C6	1.426 (2)	C19—H19B	0.9600
C7—C8	1.4405 (19)	C19—H19C	0.9600
C7—H7	0.9300	C1—H1	0.9300
			
C6—Fe1—C3	119.34 (6)	C11—C8—Fe1	129.97 (9)
C6—Fe1—C10	40.82 (6)	C10—C9—C8	108.51 (13)
C3—Fe1—C10	104.60 (6)	C10—C9—Fe1	69.45 (8)
C6—Fe1—C4	155.59 (6)	C8—C9—Fe1	70.09 (8)
C3—Fe1—C4	40.80 (6)	C10—C9—H9	125.7
C10—Fe1—C4	120.46 (6)	C8—C9—H9	125.7
C6—Fe1—C7	40.89 (6)	Fe1—C9—H9	126.3
C3—Fe1—C7	156.19 (6)	C10—C6—C7	108.43 (12)
C10—Fe1—C7	68.79 (6)	C10—C6—Fe1	69.87 (8)
C4—Fe1—C7	162.19 (6)	C7—C6—Fe1	70.01 (8)
C6—Fe1—C2	105.52 (6)	C10—C6—H6	125.8
C3—Fe1—C2	40.82 (6)	C7—C6—H6	125.8
C10—Fe1—C2	121.05 (6)	Fe1—C6—H6	125.9
C4—Fe1—C2	68.57 (6)	O1—B1—O2	114.03 (12)
C7—Fe1—C2	121.64 (6)	O1—B1—C14	123.86 (13)
C6—Fe1—C9	68.60 (6)	O2—B1—C14	122.06 (13)
C3—Fe1—C9	121.93 (6)	C16—C15—C14	121.35 (13)
C10—Fe1—C9	40.67 (5)	C16—C15—H15	119.3
C4—Fe1—C9	107.46 (6)	C14—C15—H15	119.3
C7—Fe1—C9	68.77 (6)	C4—C5—C1	108.05 (13)
C2—Fe1—C9	157.80 (6)	C4—C5—Fe1	69.37 (8)
C6—Fe1—C1	123.42 (6)	C1—C5—Fe1	69.50 (9)
C3—Fe1—C1	68.54 (6)	C4—C5—H5	126.0
C10—Fe1—C1	158.56 (6)	C1—C5—H5	126.0
C4—Fe1—C1	68.41 (6)	Fe1—C5—H5	126.7
C7—Fe1—C1	108.84 (6)	C18—C21—H21A	109.5
C2—Fe1—C1	40.69 (6)	C18—C21—H21B	109.5
C9—Fe1—C1	160.07 (6)	H21A—C21—H21B	109.5
C6—Fe1—C5	161.33 (7)	C18—C21—H21C	109.5
C3—Fe1—C5	68.38 (6)	H21A—C21—H21C	109.5
C10—Fe1—C5	157.54 (7)	H21B—C21—H21C	109.5
C4—Fe1—C5	40.47 (6)	C5—C4—C3	108.11 (13)
C7—Fe1—C5	126.07 (6)	C5—C4—Fe1	70.16 (9)
C2—Fe1—C5	68.40 (6)	C3—C4—Fe1	69.36 (8)
C9—Fe1—C5	123.65 (6)	C5—C4—H4	125.9
C1—Fe1—C5	40.67 (6)	C3—C4—H4	125.9
C6—Fe1—C8	68.88 (5)	Fe1—C4—H4	126.1
C3—Fe1—C8	159.79 (6)	C9—C10—C6	108.03 (12)
C10—Fe1—C8	68.77 (5)	C9—C10—Fe1	69.88 (8)
C4—Fe1—C8	124.87 (6)	C6—C10—Fe1	69.31 (8)
C7—Fe1—C8	41.03 (5)	C9—C10—H10	126.0
C2—Fe1—C8	158.96 (6)	C6—C10—H10	126.0
C9—Fe1—C8	40.85 (6)	Fe1—C10—H10	126.4
C1—Fe1—C8	124.36 (6)	C13—C12—C11	120.85 (13)
C5—Fe1—C8	110.09 (6)	C13—C12—H12	119.6
B1—O2—C18	107.39 (11)	C11—C12—H12	119.6
B1—O1—C17	107.07 (11)	O1—C17—C19	107.88 (12)
C12—C11—C16	118.08 (12)	O1—C17—C20	106.56 (12)
C12—C11—C8	121.46 (12)	C19—C17—C20	110.78 (14)
C16—C11—C8	120.36 (13)	O1—C17—C18	102.89 (11)
C13—C14—C15	117.52 (12)	C19—C17—C18	114.64 (12)
C13—C14—B1	121.73 (13)	C20—C17—C18	113.31 (12)
C15—C14—B1	120.50 (13)	C18—C22—H22A	109.5
O2—C18—C21	108.44 (11)	C18—C22—H22B	109.5
O2—C18—C22	106.79 (11)	H22A—C22—H22B	109.5
C21—C18—C22	110.03 (12)	C18—C22—H22C	109.5
O2—C18—C17	102.54 (10)	H22A—C22—H22C	109.5
C21—C18—C17	114.71 (12)	H22B—C22—H22C	109.5
C22—C18—C17	113.65 (12)	C4—C3—C2	108.11 (13)
C3—C2—C1	107.76 (13)	C4—C3—Fe1	69.85 (8)
C3—C2—Fe1	69.20 (8)	C2—C3—Fe1	69.98 (8)
C1—C2—Fe1	69.73 (8)	C4—C3—H3	125.9
C3—C2—H2	126.1	C2—C3—H3	125.9
C1—C2—H2	126.1	Fe1—C3—H3	125.8
Fe1—C2—H2	126.5	C17—C20—H20A	109.5
C15—C16—C11	120.74 (13)	C17—C20—H20B	109.5
C15—C16—H16	119.6	H20A—C20—H20B	109.5
C11—C16—H16	119.6	C17—C20—H20C	109.5
C6—C7—C8	107.89 (13)	H20A—C20—H20C	109.5
C6—C7—Fe1	69.10 (8)	H20B—C20—H20C	109.5
C8—C7—Fe1	70.04 (8)	C17—C19—H19A	109.5
C6—C7—H7	126.1	C17—C19—H19B	109.5
C8—C7—H7	126.1	H19A—C19—H19B	109.5
Fe1—C7—H7	126.4	C17—C19—H19C	109.5
C12—C13—C14	121.36 (13)	H19A—C19—H19C	109.5
C12—C13—H13	119.3	H19B—C19—H19C	109.5
C14—C13—H13	119.3	C2—C1—C5	107.97 (14)
C9—C8—C7	107.14 (12)	C2—C1—Fe1	69.58 (8)
C9—C8—C11	126.85 (12)	C5—C1—Fe1	69.84 (9)
C7—C8—C11	125.90 (13)	C2—C1—H1	126.0
C9—C8—Fe1	69.06 (8)	C5—C1—H1	126.0
C7—C8—Fe1	68.93 (8)	Fe1—C1—H1	126.1

### Hydrogen-bond geometry (Å, °)

**Table:**

*D*—H···*A*	*D*—H	H···*A*	*D*···*A*	*D*—H···*A*
C6—H6···C12^i^	0.93	2.86	3.6302 (19)	141
C5—H5···C6^ii^	0.93	2.62	3.538 (2)	168

Symmetry codes: (i) *x*, −*y*+3/2, *z*+1/2; (ii) *x*, −*y*+3/2, *z*−1/2.
